# Protein Adsorption at Nanorough Titanium Oxide Surfaces: The Importance of Surface Statistical Parameters beyond Surface Roughness

**DOI:** 10.3390/nano11020357

**Published:** 2021-02-01

**Authors:** Yu Yang, Steffen Knust, Sabrina Schwiderek, Qin Qin, Qing Yun, Guido Grundmeier, Adrian Keller

**Affiliations:** Technical and Macromolecular Chemistry, Paderborn University, Warburger Str. 100, 33098 Paderborn, Germany; yuyang@mail.uni-paderborn.de (Y.Y.); sknust2@campus.uni-paderborn.de (S.K.); sschwid2@mail.uni-paderborn.de (S.S.); qqqqqqinqin@gmail.com (Q.Q.); ninayun716@gmail.com (Q.Y.); g.grundmeier@tc.uni-paderborn.de (G.G.)

**Keywords:** surface topography, surface roughness, atomic force microscopy, ellipsometry, adsorption

## Abstract

The nanoscale surface topography of biomaterials can have strong effects on protein adsorption. While there are numerous surface statistical parameters for the characterization of nanorough surfaces, none of them alone provides a complete description of surface morphology. Herein, a selection of nanorough titanium oxide surfaces has been fabricated with root-mean-square roughness (*Sq*) values below 2.7 nm but very different surface morphologies. The adsorption of the proteins myoglobin (MGB), bovine serum albumin (BSA), and thyroglobulin (TGL) at these surfaces was investigated in situ by ellipsometry to assess the importance of six of the most common surface statistical parameters. For BSA adsorption, both protein film thickness and time constant of adsorption were found to scale linearly with *Sq* s. For TGL, however, the same adsorption characteristics depend linearly on the surface skewness (*Ssk*), which we attribute to the rather extreme size of this protein. Finally, a mixed behavior is observed for MGB adsorption, showing different linear correlations with *Sq* and *Ssk*. These results demonstrate the importance of a thorough morphological characterization of the surfaces employed in protein adsorption and possibly also cell adhesion studies.

## 1. Introduction

The adsorption of proteins from biological fluids represents the initial step in the response of biological systems to artificial materials [1,2,3,4]. The nature of the final biological response such as tissue integration, fibrous encapsulation, or inflammation depends strongly on the properties of the adsorbed protein film, including protein accessibility, orientation, and conformation, all of which are typically affected by the properties of the substrate surface underneath [1]. Therefore, over the last few decades, a tremendous amount of research has focused on establishing correlations between the physicochemical surface properties and the structural, functional, and biological properties of the adsorbed proteins [5,6,7,8]. While these attempts turned out rather successful with regard to the effects of surface chemistry and wettability [1,8,9,10], the role of surface topography is still not understood in detail. This is particularly true for nanorough surfaces, whose topographies have been shown to affect protein adsorption in diverse and highly complex ways [1,11,12]. In the context of the random sequential adsorption (RSA) model, it was recently shown that nanoscale surface topography can result in significantly increased protein adsorption [13]. In this particular model, steric hindrance between adsorbing proteins prevents the complete coverage of the surface. For a flat surface, this jamming limit occurs at a surface coverage of only about 55%. Nanoscale surface protrusions, however, can result in reduced steric hindrance and thus an increase in surface coverage, while the opposite may be observed for depressions [13]. The magnitude of this effect depends strongly on the curvature of the nanotopographic features and is thus particularly important with regard to colloidal nanoparticles and films thereof, which come in a large variety of different sizes, shapes, and curvatures [14,15]. However, nanoscale surface topography may also hinder lateral surface diffusion of adsorbed or adsorbing proteins [16] and even affect adsorption-induced protein unfolding and denaturation [17].

Oxides represent a particularly important class of biomaterials as they are found in many orthopedic and dental implants, either in the form of ceramic materials, such as alumina [18] and zirconia [19], or as native surface oxides on metallic implants, such as titanium [20] and tantalum [21]. Consequently, the effect of the nanoscale topography of such oxide surfaces on the adsorption of various proteins has been the focus of numerous studies [22,23,24,25,26,27,28,29,30,31,32]. For instance, Rechendorff et al. investigated the adsorption of bovine serum albumin (BSA) and human plasma fibrinogen at oxidized tantalum surfaces with a root-mean-square (RMS) surface roughness (*Sq*) between 2 and 33 nm [22]. Within this *Sq* range, the authors observed a continuous increase in the mass density of adsorbed fibrinogen up to about 70%. A similar, yet smaller, increase in adsorbed mass of about 30% was observed for BSA. In a similar experiment, Rockwell et al. studied the adsorption of BSA and bovine plasma fibrinogen on an oxidized titanium film with a *Sq* gradient ranging from 1 to 16 nm [25]. For both proteins, the authors observed a 50% increase in the adsorbed protein mass in the *Sq* range from ~2 to ~8 nm. For larger *Sq* values, the amount of adsorbed proteins saturated. On the other hand, Cai et al. investigated the adsorption of BSA and human plasma fibrinogen at oxidized titanium films with *Sq* ranging from 2 to 21 nm [23]. Surprisingly, however, the authors did not observe any significant differences in the adsorption of both proteins in these particular experiments, despite their surfaces having *Sq* values comparable to those used by Rockwell et al.

A general problem faced by such studies is the morphological description of the nanorough surfaces. While there are many statistical parameters that can be used to describe a rough surface, none of these parameters provides a complete description of the full surface topography. For instance, the widely employed RMS surface roughness *Sq*, which can be readily computed from atomic force microscopy (AFM) images by virtually all commercial and open-source AFM software solutions, is nothing more than the second-order moment of the surface height distribution and, thus, a measure of the fluctuations of surface height values around the mean height [33]. Therefore, surfaces with very different topographies can have identical *Sq* values. A more detailed description of a given surface thus needs to consider also other, higher-order moments or even the full power spectrum [33]. Indeed, a few recent studies have shown that the physical behavior of rough surfaces, for instance during thin film deposition, depends non-trivially on higher-order moments of the surface height distribution such as surface skewness (*Ssk*) and kurtosis (*Sku*), which thus need to be carefully characterized in detail [34,35,36]. 

In this work, we therefore sought to identify possible correlations between different surface statistical parameters and the adsorption of three rather different globular proteins. To this end, we have prepared a selection of titanium thin films whose oxidized surfaces have almost identical chemical compositions as verified by X-ray photoelectron spectroscopy (XPS) but very different nanorough topographies with low *Sq* values ranging from 0.2 to 2.7 nm. Using AFM to characterize these model surfaces, six of the most widely used parameters for the statistical description of rough surfaces were determined. Then, the adsorption of three globular proteins with different molecular weights, sizes, and charges at these surfaces was studied under identical conditions in situ by ellipsometry. Remarkably different behaviors were observed for the proteins under study. In particular, for bovine serum albumin (BSA), we found that both adsorption kinetics and the saturated thickness of the adsorbed protein film exhibit weak linear correlations with the RMS roughness *Sq*, whereas the same characteristics were observed to scale linearly with surface skewness *Ssk* in the case of thyroglobulin (TGL). Myoglobin (MGB), on the other hand, showed a mixed behavior. Here, the protein film thickness depends linearly on *Sq*, while the time constant of adsorption scales with *Ssk*. Our results thus demonstrate the necessity of a complete morphological characterization of the nanorough surface topographies in protein adsorption experiments. 

## 2. Materials and Methods 

### 2.1. Preparation of Titanium Oxide Model Surfaces

Titanium thin films have been grown on epi-ready p-doped Si(100) wafers (Siegert Wafer, Aachen, Germany) by magnetron sputter deposition (Ion’X-2” UHV from Thin Film Consulting, Grafenberg, Germany, with a BDS-HF 300 AFP generator from BDISCOM SRL, Vellezzo Bellini, Italy) using a high-purity titanium target (purity 99.995%, EVOCHEM, Offenbach am Main, Germany) as previously described [17]. Five different sample types have been fabricated using the deposition conditions listed in Table 1. In addition to the different deposition conditions, for one of the samples (0.07-40-r), we have used a nanorippled silicon substrate. This substrate was pretreated as previously described by 500 eV Ar^+^ irradiation at an oblique angle of incidence, which resulted in a regular quasi-sinusoidal ripple pattern spontaneously forming on the silicon surface with a periodicity and a peak-to-peak height of about 30 nm and 1.5 nm, respectively [17].

### 2.2. XPS Characterization of the Titanium Oxide Model Surfaces

To characterize the surface composition of the titanium-coated substrates, XPS was performed in an ESCA+ facility (Oxford Instruments, Taunusstein, Germany) at a base pressure <4.0 × 10^−10^ mbar using monochromatic Al Kα radiation (1486.7 eV). The samples were measured without neutralization, calibrating the spectra to the C 1s signal (at 284.6 eV) of adventitious carbon. The spectra were collected at a take-off angle of 30° with respect to the surface using a pass energy of 100 eV and a step size of 0.2 eV for survey spectra and a pass energy of 20 eV and a step size of at least 0.1 eV for core level spectra.

### 2.3. AFM Characterization of the Titanium Oxide Model Surfaces

The fabricated sample surfaces were characterized by AFM in air using an Agilent 5500 and a JPK Nanowizard 3 AFM operated in intermittent contact mode and HQ:NSC18/AlBS cantilevers (MikroMasch, Wetzlar, Germany) with a nominal tip radius <8 nm. In order to minimize the impact of artefacts resulting from tip-to-tip variations and tip wear, cantilevers were frequently replaced by fresh ones during the course of the AFM measurements, so that the results of the statistical analyses represent not only averages of several AFM images but also of several cantilevers. All images were recorded with a scan size and a resolution of 2 × 2 µm² and 1024 × 1024 pixels, respectively, and analyzed using Gwyddion open source software [37]. To this end, the images were preprocessed by mean plane subtraction, row alignment using the median, and subtraction of a third-degree polynomial. The height values of the images were subsequently normalized by setting the height minimum to zero. Statistical analyses were then carried out using the Statistical Quantities, the Statistical Functions, and the Fractal Dimension tools of Gwyddion. See the Gwyddion user guide for details [38].

### 2.4. In Situ Ellipsometry Investigation of Protein Adsorption Kinetics

Lyophilized MGB from equine heart, BSA, and TGL from bovine thyroid were purchased from Sigma Aldrich, Steinheim, Germany, and dissolved at concentrations of 1 (MGB, TGL) and 10 mg/mL (BSA) in phosphate-buffered saline (PBS, VWR, Hannover, Germany) containing 137 mM sodium chloride, 2.7 mM potassium chloride, and 10 mM phosphate buffer at pH 7.4. A higher concentration of BSA was employed because no BSA adsorption could be detected at 1 mg/mL. This may be attributed to the strong tendency of BSA to undergo conformational changes during adsorption [17]. A lower concentration will lead to a slower arrival of adsorbing BSA molecules and, thus, stronger subsequent spreading at the sample surfaces [39], which, in turn, will result in a thicknesses of the adsorbed BSA films below the detection limit of the ellipsometry setup.

Protein adsorption at the different surfaces was assessed by in situ ellipsometry as previously described [17] using an auto-nulling ellipsometer (Ep3, Accurion GmbH, Göttingen, Germany) with a 658 nm laser as light source. Prior to each experiment, the surface of the corresponding substrate was thoroughly washed with ethanol and dried in a stream of ultrapure air. This mild cleaning protocol was chosen over harsher procedures, such as piranha cleaning, in order to avoid acid-induced damage and delamination of the thin titanium films. The protein-containing buffer solutions were injected into the flow cell at 100 µL/min after 10 min of equilibration in protein-free buffer. After 30 min of continuous injection to ensure that the whole volume of the flow cell was replaced, the pump was stopped and the measurements were continued under static conditions until the flow cell was flushed with protein-free buffer at 100 µL/min.

The thickness of the adsorbed protein layer was derived by modelling the optical properties of the substrate in contact with the protein-containing solution. To this end, a three-layer model was employed. The first layer consisted of the effective substrate based on a unique n/k-model of each substrate surface taken before protein adsorption as described in detail in [17]. This n/k-model implicitly accounts for possible variations in titanium film thickness, oxide layer thickness, and surface topography. The second layer consisted of the adsorbed protein film, whose reflective index was modelled using a Cauchy dispersion function [40]:

(1) $$n\left(\lambda \right)=A+\frac{B}{\lambda^{2}}+\frac{C}{\lambda^{4}}$$

with the parameters *A* = 1.42, *B* = 0.01 μm^−2^, and *C* = 0 μm^−4^ taken from literature [41]. For the third layer, H_2_O at 21.5 °C was used as the ambient material. The average RMS error (RMSE) for the modelled experiments ranged from 0.95 to 1.95. The time constant of adsorption *τ* was obtained by fitting the time-dependent adsorption curves with the exponential function:

(2) $$\mathrm{thickness}=C\left(1-e^{-\frac{t}{\tau }}\right),$$

where *C* was used as a fit parameter (see Figures S3–S7).

## 3. Results and Discussion

### 3.1. Characterization of the Titanium Oxide Model Surfaces

**Figure 1:**
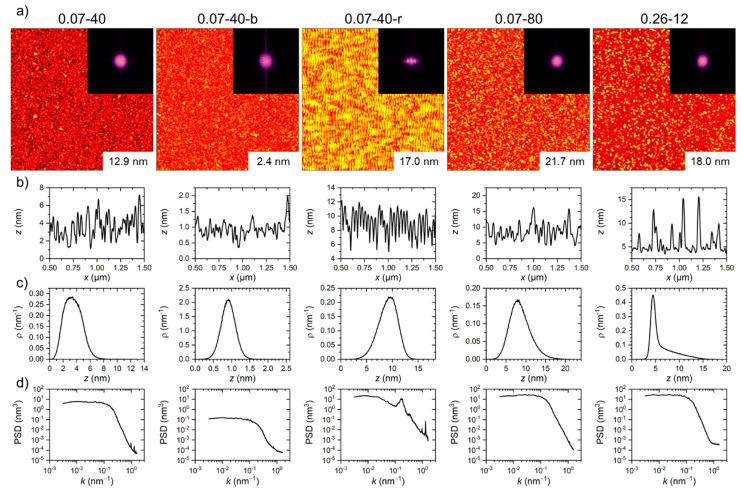
Representative AFM images (2 × 2 µm², ranges of the height scales as given in the images) with corresponding FFTs (**a**), horizontal height profiles taken in the center of the images (**b**), height distribution functions (**c**), and one-dimensional (horizontal) PSD functions (**d**) of the different titanium oxide samples.

The fabricated surfaces were first thoroughly characterized by XPS and AFM. XPS confirmed that the surfaces of the different titanium films have very similar compositions and oxidation states (see Figures S1 and S2 and Tables S1 and S2), so that effects of surface chemistry on protein adsorption should be negligible. AFM on the other hand revealed pronounced differences in surface morphology. Figure 1a shows AFM images of all samples together with their corresponding two-dimensional fast Fourier transforms (FFTs). The latter reveal that four of the model surfaces are perfectly isotropic. Only the surface of sample 0.07‑40‑r, which was grown on the nanorippled substrate, shows a strong degree of anisotropy. The height profiles shown in Figure 1b further reveal that the five samples have very different surface topographies. While sample 0.07-40-r predictably shows a very periodic surface modulation with an overlaying long-range roughness [42], sample 0.07-40-b has a random rough morphology and is rather flat. This is because of the applied bias potential during titanium deposition, which results in very smooth and highly conformal films that replicate the substrate topography almost perfectly [17]. In the absence of a bias potential, however, a much rougher surface is obtained under otherwise identical conditions (sample 0.07-40). Here, the total height variation within a 1 µm profile is about 6 nm, compared to about 1.5 nm for sample 0.07-40-b. For an increasing film thickness, this height variation is further increased as predicted by dynamic scaling theory [34,43] and frequently observed in various growth processes [44,45]. Consequently, for sample 0.07-80, the maximum height variation observed in the height profile in Figure 1b has reached a value of more than 10 nm. Increasing the deposition rate by a factor of more than three, on the other hand, resulted in a very different surface morphology at a similar film thickness that is characterized by rather pronounced spikes on top of a closed titanium film. 

These morphological differences can be assessed even better in the height distribution functions shown in Figure 1c. The height distribution functions were normalized so that the area under each curve equals 1. Obviously, sample 0.07-40-b has a much narrower height distribution function than sample 0.07-40, as could already be expected based on the height profiles in Figure 1b. While these two height distribution functions appear more or less symmetric, the height distribution function of sample 0.26-12 is characterized by a rather narrow peak located around 5 nm height and a broad shoulder that extends to heights up to about 15 nm. The height distribution functions of samples 0.07-40-r and 0.07‑80 are very interesting as well. As can be seen at first glance in Figure 1c, these height distribution functions are asymmetric, with that of sample 0.07‑40‑r and 0.07-80 having a notable tail toward smaller and larger heights, respectively. 

Finally, Figure 1d shows the one-dimensional power spectral density (PSD) functions of the different sample surfaces. The overall shape of the PSD functions is characterized by a flat plateau and a falling slope at low and high spatial frequencies, respectively, which indicates that the surface roughness is correlated at short distances but not at large ones. Despite the similar PSD shapes, distinct differences between samples are observed. The smoothest sample 0.07-40-b also has the lowest PSD intensities of all samples, which is reasonable because the RMS surface roughness *Sq* is proportional to the square root of the integral of the PSD [46]. Furthermore, sample 0.07-40-r displays a pronounced correlation peak at a spatial frequency *k* ~ 2 × 10^−1^ nm^−1^, which corresponds to the ripple periodicity *l* = 2π/*k* ~ 30 nm.

From such AFM images, six surface statistical parameters have been calculated (see Table 2 and Table S3 for details). The RMS surface roughness *Sq* is a measure of the fluctuations of surface heights around the mean surface. It is closely related to the arithmetic surface roughness *Sa* (see Table S3), which was calculated as well for the sake of completeness. Both parameters show that sample 0.07-40-b has by far the smoothest surface with *Sq* ~ 0.26 nm (Table 2). Despite their very different surface topographies, samples 0.07-40 and 0.07-40‑r have rather similar roughness values of *Sq* ~ 1.4 and 1.5 nm, respectively. A similar observation is made for samples 0.07-80 and 0.26-12, which have *Sq* values of ~ 2.67 and ~ 2.58 nm, respectively. The same trends are also observed in the roughness factor *r*, defined as the ratio of the real three-dimensional surface area and the projected two-dimensional surface area, which in this case is represented by the scan size of the AFM image. The fact that so similar first- and second-order surface roughness parameters are obtained for very different surface morphologies clearly demonstrates the need for additional higher-order parameters to characterize the morphology of the titanium oxide surfaces.

The surface skewness *Ssk* is the third-order standardized moment of the surface height distribution and measures the symmetry of the height distribution function [33]. It has positive values for samples 0.07-40, 0.07-40‑b, 0.07-80, and 0.26-12 (see Table 2), indicating different degrees of asymmetry with the distributions having tails toward larger heights. Here, the strongest asymmetry is observed for sample 0.26-12, in agreement with the visual inspection of the corresponding height distribution function in Figure 1c. Interestingly, however, sample 0.07-40 exhibits the second largest asymmetry in its height distribution function, which is not as easily recognizable in Figure 1c, because it is unusually broad. Finally, sample 0.07-40‑r has the only surface with a negative skewness, which indicates a height distribution function with a tail toward smaller heights, as can clearly be seen in Figure 1c. This can be attributed to nonlinear effects occurring during the self-organized formation of the ripple pattern on the substrate surface [47].

The fourth-order standardized moment of the surface height distribution is the kurtosis *Sku*, which measures the sharpness of the height distribution function [33]. In general, *Sku* = 3 corresponds to a Gaussian height distribution function [33]. This is observed only for sample 0.07-40-r, which has a kurtosis of exactly 3. This can be attributed to the quasi-sinusoidal surface modulation of the ripple pattern. For all the other samples, *Sku* > 3, indicating that the height distribution functions are leptokurtic. This would correspond to surfaces with spike-like depressions and elevations. The largest *Sku* of about 7 is obtained for sample 0.07‑40 (see Table 2), which also has the broadest height distribution function as can be seen in Figure 1c. Samples 0.07-40-b and 0.26-12 have a smaller *Sku* > 4, whereas for sample 0.07-80, *Sku* ~ 3.7. 

Finally, we have also calculated the fractal dimension *D* of each sample surface. The fractal dimension is not based on the moments of the height distribution but rather represents a measure of the complexity of the surface’s morphology and can be derived either directly using for instance the cube counting method as done here or from the power spectrum [33,48]. Both methods have been evaluated and are compared in Table S4. For all sample surfaces, the power spectrum-based method yielded values that are about 25 to 50% smaller than those of the cube counting method and have larger standard deviations. Since it was observed previously that the power spectrum-based method shows lower performance in the analysis of microscopy images [49], we have used only the values obtained by the cube counting method in the following analyses. 

The fractal dimensions of the different samples in Table 2 are very similar with values around 2.5, despite the pronounced differences in the other statistical parameters. Since the fractal dimension *D* is related to the slope of the power spectrum in a log-log plot [33], this indicates that the two-dimensional power spectra of the different samples have similar shapes, which appears reasonable based on one-dimensional PSD functions and the 2D FFTs shown in Figure 1. This is further supported by the *D* values obtained using the power spectrum-based method, which are rather similar as well (see Table S4).

### 3.2. Effect of Titanium Oxide Surface Morphology on Protein Adsorption

The adsorption of the three globular proteins MGB, BSA, and TGL at the titanium oxide model surfaces was studied in situ by ellipsometry. Titanium oxide has an isoelectric point of about 3.5 and is thus negatively charged at neutral pH [50]. Protein adsorption at the titanium oxide surfaces under physiological conditions will thus be governed by electrostatic interactions. However, van-der-Waals interactions may play a role as well, as titanium oxide also has a comparatively large Hamaker constant [50]. MGB is a 17.8 kDa protein with a small positive net charge at pH 7.4 [17]. BSA, on the other hand, has a molecular weight of 66.5 kDa and carries an intermediate negative net charge under the same conditions [17]. Finally, TGL is a much larger protein with a molecular weight of 660–690 kDa and a strong negative net charge at physiological pH [17]. The adsorption of these proteins was studied under the same conditions as in previous experiments [17]. In particular, PBS buffer at pH 7.4 was chosen as the ambient medium to mimic physiological fluids. The protein concentrations were selected to yield clearly detectable thickness values for the protein films adsorbed at the substrate surface with the lowest RMS roughness, i.e., sample 0.07-40-b.

**Figure 2:**
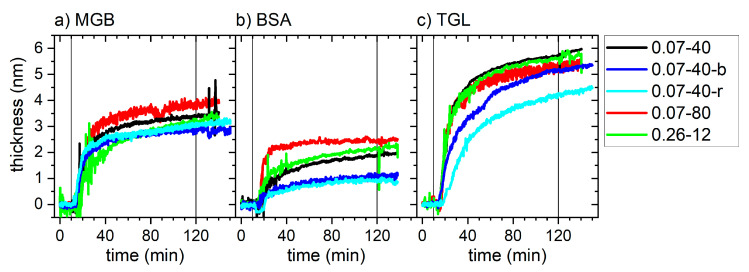
Protein layer thickness for MGB (**a**), BSA (**b**), and TGL (**c**) at the different titanium oxide model surfaces as measured by ellipsometry. The vertical lines indicate the injection of protein-containing buffer solution and the flushing of the flow cell with protein-free buffer, respectively.

Figure 2 shows the thickness of the adsorbed protein films as a function of incubation time. In general, a rapid increase in film thickness is observed upon protein injection, with subsequent saturation. While rather strong differences are observed between the different proteins, owing to their very different sizes [17], the different model surfaces behave rather similar. In this regard, the strongest deviations between individual model surfaces are observed for BSA and TGL, whereas for MGB, adsorption kinetics and final film thickness are more similar for the different surfaces. Note that flushing with protein-free buffer did not lead to visible desorption for any of these surfaces and proteins, which is indicative of irreversible adsorption.

**Figure 3:**
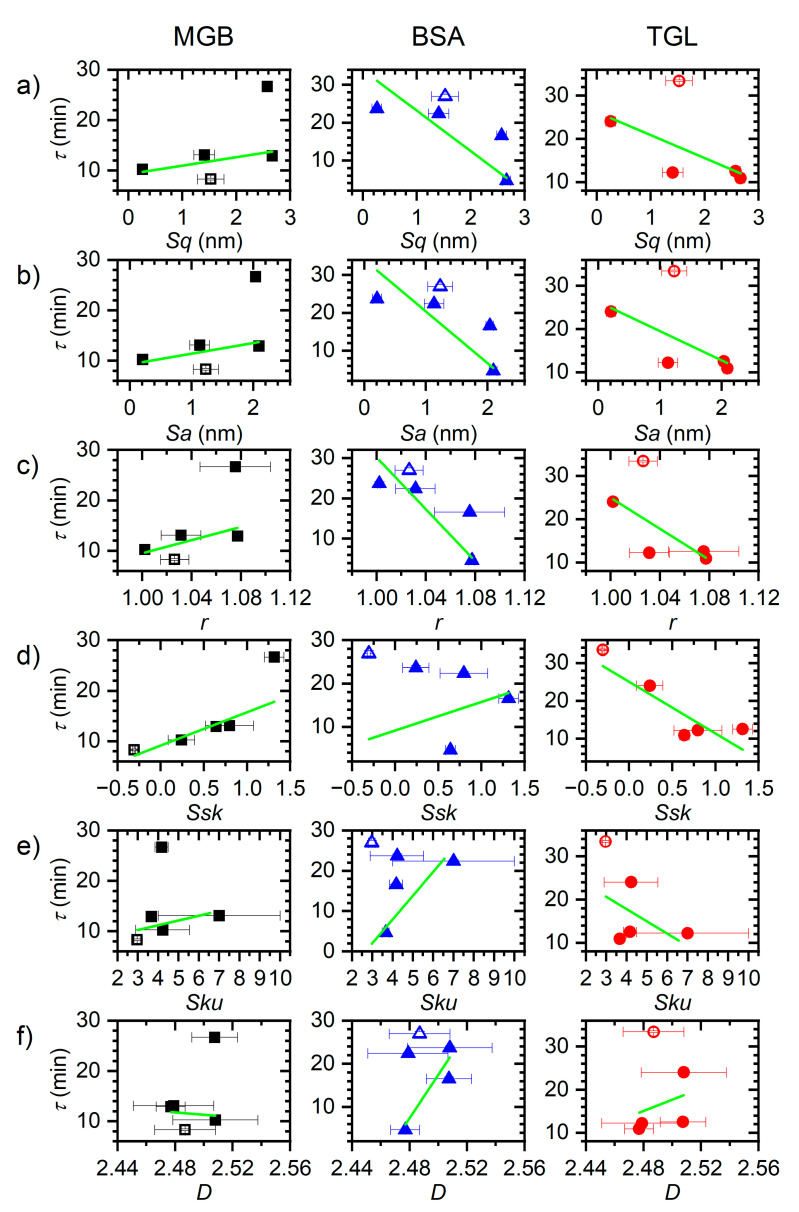
Time constant of adsorption *τ* of MGB, BSA, and TGL determined by fitting the ellipsometry data in Figure 2 (see Figures S3–S7) versus RMS surface roughness *Sq* (**a**), arithmetic surface roughness *Sa* (**b**), roughness factor *r* (**c**), skewness *Ssk* (**d**), kurtosis *Sku* (**e**), and fractal dimension *D* (**f**). Error bars in *x* direction reflect the standard deviations given in Table 2, whereas error bars in *y* direction represent the errors of the exponential fits. The nanorippled surfaces (0.07-40-r) are indicated by the empty symbols. The solid green lines represent linear fits to the data.

The ellipsometry measurements in Figure 2 were quantitatively analyzed by fitting them with an exponential function (see Materials and Methods and Figures S3–S7) in order to extract the time constant of adsorption. While most of those fits did yield comparatively high *R*^2^ values >0.9, some of them could not reproduce the full dynamics of the experimental data. In particular, at several instances, it is observed that the thickness of the adsorbed protein layer is still slightly increasing in the plateau phase. This particular observation can be attributed to the fact that the ellipsometry measurements were conducted under static conditions, so that upon starting the pump for flushing the flow cell with protein-free buffer after the experiment, the flow cell was subjected to a second injection of protein-containing solution that was trapped during the static measurement in the connected tubing. The resulting increase in protein concentration obviously led to some additional yet small adsorption at the surface, possibly followed by further structural rearrangements in the adsorbed protein layer during flushing. Furthermore, it should be noted that perfect fits of protein adsorption data usually require more complicated models [51]. Nevertheless, we used the time constants obtained from these fits as a first-order approximation to compare adsorption kinetics at the different surfaces [50]. In Figure 3, these time constants are plotted versus the different surface statistical parameters of the individual samples listed in Table 2. As can be seen, positive as well as negative roughly linear correlations can be observed for certain protein-parameter combinations, whereas others appear not to show any pronounced dependencies at all. Remarkably, for several of those combinations, the nanorippled surfaces 0.07‑40-r (empty symbols) do not follow the overall trends of the other sample surfaces.

**Figure 4:**
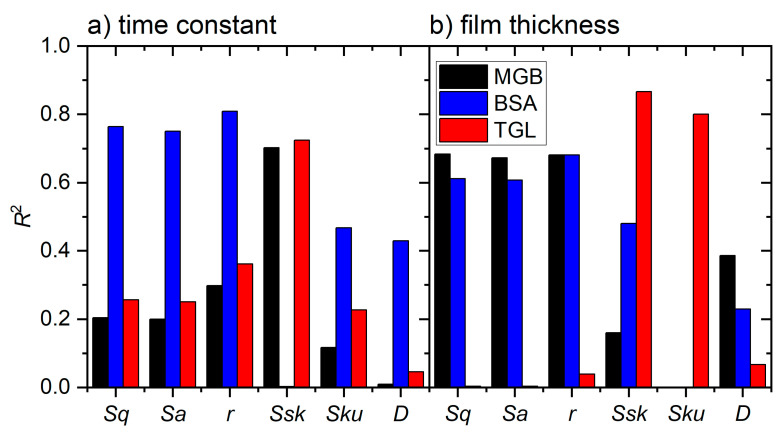
*R*² values of the linear fits to the time constant data in Figure 3 (**a**) and the film thickness data in Figure 5 (**b**). The individual *R*² values are listed in Tables S5 and S6, respectively.

In order to determine which of the linear correlations in Figure 3 are the strongest, we have fitted all the data with linear functions and evaluated the quality of the fits based on their *R*² values. The individual *R*² values are plotted in Figure 4a and reveal surprisingly strong differences between the selected proteins. In particular, only one protein, namely BSA, shows linear correlations with 0.75 < *R*² < 0.85 between the time constant of adsorption and the established surface roughness parameters *Sq*, *Sa*, and *r*. These correlations are negative (see Figure 3a–c), which implies that BSA adsorption proceeds faster at a rougher surface. This can be attributed to the larger number of surface sites available for protein binding. The fact that almost identical correlations are observed between the time constant and each of the three parameters *Sq*, *Sa*, and *r* is not surprising either, as these parameters are closely related indeed [33]. In contrast, however, MGB and TGL do not show any significant correlations between these parameters and their time constants of adsorption. Rather, both proteins show weak correlations (*R*² ~ 0.7) with the surface skewness *Ssk*, which are harder to rationalize as the skewness is simply a measure of the symmetry of the height distribution function. Furthermore, MGB and TGL show different correlations. While a positive linear correlation is observed for MGB, the time constant of TGL adsorption follows a negative correlation (see Figure 3d).

**Figure 5:**
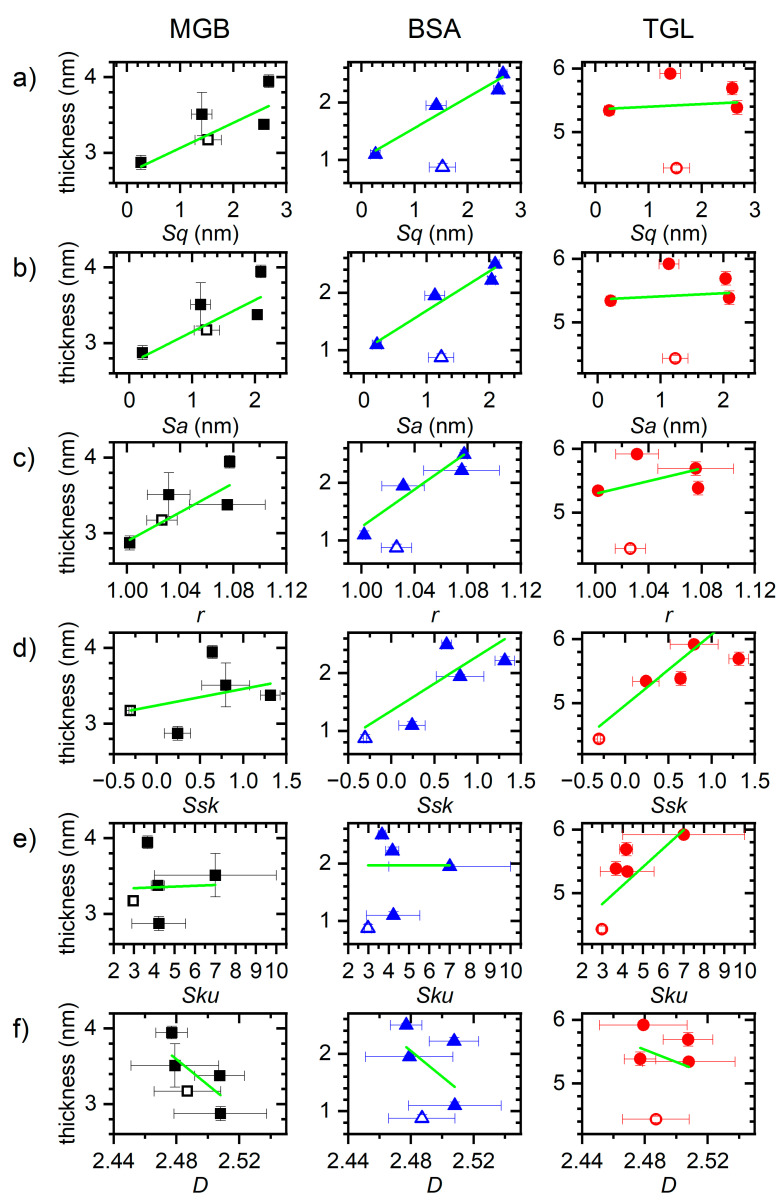
Thickness of the irreversibly adsorbed MGB, BSA, and TGL films determined from the ellipsometry data in Figure 2 versus RMS surface roughness *Sq* (**a**), arithmetic surface roughness *Sa* (**b**), roughness factor *r* (**c**), skewness *Ssk* (**d**), kurtosis *Sku* (**e**), and fractal dimension *D* (**f**). Error bars in *x* direction reflect the standard deviations given in Table 2, whereas error bars in *y* direction represent the standard deviations from averaging the thickness values recorded in the last 10 min of the experiment. The nanorippled surfaces (0.07-40-r) are indicated by the empty symbols. The solid green lines represent linear fits to the data.

At this point, it should be mentioned that protein adsorption at solid surfaces is a highly complex phenomenon influenced by numerous protein, solution, and surface properties. The adsorption kinetics of a given protein can be expected to be governed mostly by protein concentration and surface chemistry, i.e., charge and hydrophobicity, while surface topography probably plays only a minor, modulating role. Most previous studies thus rather considered the amount of adsorbed protein at saturation as a more sensitive measure to identify the effects of different surface properties [17,22,23,24,25,26,52,53,54]. Therefore, we next determined the saturated thicknesses of the irreversibly adsorbed protein films after flushing by averaging the data points recorded in the last 10 min of the experiments. As can be seen in the plots shown in Figure 5, several roughly linear correlations are obtained for different surface statistical parameters. In fact, only the fractal dimension *D* (Figure 5f) does not seem to show any correlation for either protein. All the other parameters in Figure 5a–e, however, appear to have a positive correlation with the saturated protein layer thickness, which can be more or less pronounced depending on the protein and the actual parameter. Again, for several protein-parameter combinations in Figure 5, the nanorippled surfaces 0.07-40-r (empty symbols) do not follow the general trend of the other sample surfaces and show a lower protein film thickness than would otherwise be expected based on their statistical parameters. This in particular concerns the parameters *Sq*, *Sa*, and *r*.

Based on the *R*² values of the linear fits to the data in Figure 5, MGB and BSA appear to behave rather similar, with the largest *R*² values being obtained for the correlations between protein film thickness and the second-order moments *Sq* and *Sa*, as well as for the roughness factor *r* (see Figure 4b). For all these parameters, *R*² values between 0.6 and 0.7 are obtained for both proteins, indicating weak linear correlations. Several studies have reported positive correlations between surface roughness (*Sq* or *Sa*) and the amount of adsorbed proteins [22,25,26,28,52,53,54]. In the case of BSA adsorption, the correlations obtained in Figure 4b for the protein film thickness are very similar to the ones found in Figure 4a for the time constant of adsorption. This implies that a rougher surface leads to faster BSA adsorption and a thicker protein film. This can be rationalized by the interplay between adsorption kinetics and the degree of adsorption-induced protein conformational changes, as faster adsorption in general results in less spreading of the protein at the surface, so that on average a thicker film is formed [39]. This effect can be expected to be particularly pronounced for BSA, which is well known to undergo strong denaturation during adsorption [17]. For MGB on the other hand, it appears that protein film thickness is depending on *Sq*, *Sa*, or *r*, while adsorption kinetics are mostly influenced by *Ssk*. This indicates that there is no or only weak MGB denaturation during adsorption, which agrees with previous AFM-based investigations [17]. In this case, a larger surface roughness does not result in faster adsorption but only in a thicker protein film, presumably because of reduced steric hindrance leading to a higher surface coverage beyond the jamming limit of a flat surface [13]. At the same time, an increase in surface skewness *Ssk*, which is equivalent to the appearance of surface protrusions, slows down MGB adsorption. This may hint at the importance of lateral surface diffusion of adsorbed proteins during adsorption, which is hindered by protruding topographical features [16].

**Figure 6:**
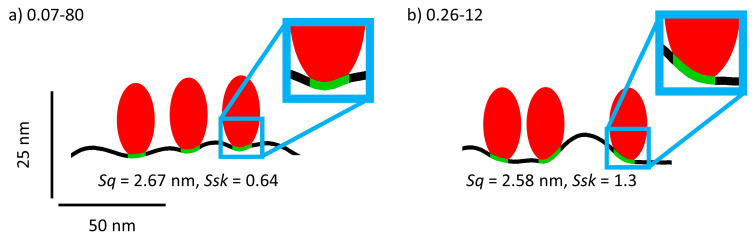
Schematic representation of TGL (red) adsorbed to the surfaces of samples 0.07-80 (**a**) and 0.26‑12 (**b**), respectively. Both surfaces have rather similar *Sq* but different *Ssk* values as indicated in the figure. The height profiles were taken from corresponding AFM images. The size and shape of TGL were estimated based on its hydrodynamic radius of 8.6 nm. The areas of contact between TGL and the surface profile are highlighted in green. Note that the vertical axis has been enhanced by a factor of two to better visualize the comparably small differences between the two surface morphologies.

For TGL adsorption, the situation is quite different. Here, comparatively strong linear correlations with 0.8 < *R*² < 0.9 are obtained between the thickness of the adsorbed protein film and both parameters *Ssk* and *Sku*. For this protein, there is essentially no correlation at all with the second-order moments *Sq* and *Sa* and the roughness factor *r*, all of which have yielded *R*² << 0.1 (see Table S6). This is particularly noteworthy as a similar yet slightly weaker correlation is observed also between *Ssk* and the time constant of adsorption (see Figure 4a). This leads to the rather surprising insight that both TGL adsorption kinetics and adsorbed TGL film thickness scale with the skewness *Ssk* of the sample surface and not with the surface roughness or effective surface area. The most obvious explanation for this peculiar behavior of TGL lies in its rather extreme size. With a molecular weight of more than 0.5 MDa and a diameter of more than 15 nm [17], it appears rather reasonable that steric hindrance will render a large fraction of the actual surface area of the rougher surfaces inaccessible to this protein, so that it will not display strong correlations with *Sq*, *Sa*, or *r*. Rationalizing the strong sensitivity of this protein for *Ssk* and *Sku* is more difficult. A large kurtosis *Sku* > 3 means that the surface has a topography composed of spike-like elevations and/or depressions. A large positive skewness on the other hand means that the majority of surface height values are located below the mean height. Since the thickness of the adsorbed TGL film correlates positively with both values, we can conclude that TGL adsorption is enhanced at surfaces composed of few but high spike-like elevations. Presumably, such surfaces allow the large TGL protein to maximize its contact area with the surface by adsorbing at locations between the spikes that enable contact with the rather smooth surface below as well as with the spike sidewalls (see Figure 6). In this way, a larger fraction of the effective surface area will be accessible to the protein, while steric hindrance between neighboring proteins may be reduced. This in turn may accelerate protein adsorption (see Figure 3d and Figure 4a) and lead to larger surface coverage exceeding the jamming limit, which will be detected as an increase in average film thickness (see Figure 4b and Figure 5d,e). While this explanation is rather speculative at the current stage, future experiments with a range of rationally designed surfaces produced by lithographic techniques may shed more light on the mechanisms involved.

## 4. Conclusions

In summary, we have investigated the adsorption of the three globular proteins MGL, BSA, and TGL at a selection of titanium oxide surfaces with almost identical chemical compositions but very different surface topographies. By thoroughly characterizing the morphologies of these surfaces by AFM, we were able to screen for possible correlations between a selection of the most widely employed surface statistical parameters and the time constants of adsorption as well as the thickness of the irreversibly adsorbed protein films. Rather different behaviors were identified for the three proteins. For BSA, adsorption was found to proceed faster at surfaces with higher RMS roughness *Sq* and result in a larger thickness of the protein film at saturation. This indicates that BSA adsorption at these titanium oxide surfaces is mostly governed by the number of available adsorption sites and involves significant protein denaturation and spreading. For MGB, however, only the protein film thickness was observed to scale with *Sq*, while the time constant of adsorption followed a linear correlation with surface skewness *Ssk*. This may indicate that MGB adsorption involves the lateral diffusion of adsorbed proteins but no protein denaturation. In the case of TGL, it was found that adsorption is mainly influenced by the surface skewness, with larger *Ssk* values resulting in faster adsorption and thicker protein films. This, we attribute to the rather extreme size of this protein, which can adsorb more readily at surfaces with sparse, spike-like protrusions as these allow it to maximize its contact area with the surface. 

These rather surprising observations clearly demonstrate that studies investigating the effects of different surface topographies on protein adsorption require a detailed and thorough characterization of the surface morphologies of the employed substrates. The same also holds true for the evaluation of different physical and chemical surface treatments that may modify the original surface morphology in almost indiscernible ways. Furthermore, since surface topography not only affects the amount of adsorbed proteins but also the biological properties of the adsorbed protein films, e.g., because of differences in protein denaturation, we anticipate that similar surface morphology-specific, rather than surface roughness-related effects, may also be observed in cell adhesion experiments.

## Figures and Tables

**Table 1:** Sample and thin film preparation conditions.

Sample ID	0.07-40	0.07-40-b	0.07-40-r	0.07-80	0.26-12
Substrate	flat	flat	rippled	flat	flat
Deposition rate (Å/s)	0.07	0.07	0.07	0.07	0.26
Deposition time (min)	40.0	40.0	40.0	80.0	12.2
Applied bias potential (V)	0	−10	−10	0	0

**Table 2:** Surface statistical parameters calculated from the AFM images of the different samples. Values represent averages over four AFM images taken at different sample surfaces with standard deviations given as errors.

Sample ID	0.07-40	0.07-40-b	0.07-40-r	0.07-80	0.26-12
*Sq* (nm)	1.4 ± 0.2	0.26 ± 0.09	1.5 ± 0.3	2.67 ± 0.08	2.58 ± 0.09
*Sa* (nm)	1.1 ± 0.2	0.21 ± 0.07	1.2 ± 0.2	2.09 ± 0.06	2.04 ± 0.06
*r*	1.03 ± 0.02	1.00 ± 0.01	1.03 ± 0.01	1.08 ± 0.01	1.08 ± 0.03
*Ssk*	0.8 ± 0.3	0.24 ± 0.15	−0.31 ± 0.02	0.64 ± 0.06	1.3 ± 0.1
*Sku*	7 ± 3	4.2 ± 1.3	3.0 ± 0.2	3.7 ± 0.2	4.2 ± 0.3
*D*	2.48 ± 0.03	2.51 ± 0.03	2.49 ± 0.02	2.48 ± 0.01	2.51 ± 0.02

## Data Availability

The data presented in this study are available on request from the corresponding author.

## Supplementary Materials

The following are available online at https://www.mdpi.com/2079-4991/11/2/357/s1, Figure S1. Ex situ XPS survey of the various titanium coated substrates, Figure S2. Ex situ XPS Ti 2p high-resolution spectra of the various titanium coated substrates, Figure S3. Protein layer thickness for MGB, BSA, and TGL at surface 0.07-40 as measured by ellipsometry, Figure S4. Protein layer thickness for MGB, BSA, and TGL at surface 0.07-40-b as measured by ellipsometry, Figure S5. Protein layer thickness for MGB, BSA, and TGL at surface 0.07-40-r as measured by ellipsometry, Figure S6. Protein layer thickness for MGB, BSA, and TGL at surface 0.07-80 as measured by ellipsometry, Figure S7. Protein layer thickness for MGB, BSA, and TGL at surface 0.26-12 as measured by ellipsometry, Table S1. XPS quantification results, Table S2. Results of the Ti 2p deconvolution, Table S3. Calculation of moment-based surface statistical parameters, Table S4. Comparison of the fractal dimension determined by the cube counting method and from a linear fit to the slope of the power spectra in the log-log plot, Table S5. R² values of the linear fits to the time constant data shown in Figure 3, Table S6. R² values of the linear fits to the protein layer thickness data shown in Figure 5.
