# Supplementary material for: Protein Adsorption at Nanorough Titanium Oxide Surfaces: The Importance of Surface Statistical Parameters beyond Surface Roughness
Source: Nanomaterials (Basel). 2021 Feb 1;11(2):357. doi: 10.3390/nano11020357 (PMC7912717; doi:10.3390/nano11020357)
Supplement: Supplementary file 1 [file nanomaterials-11-00357-s001.pdf]

nanomaterials

MDPI

Supplementary Materials

Protein Adsorption at Nanorough Titanium Oxide Surfaces: The Importance of Surface Statistical Parameters beyond Surface Roughness

Yu Yang, Steffen Knust, Sabrina Schwiderek, Qin Qin, Qing Yun, Guido Grundmeier and Adrian Keller *

Technical and Macromolecular Chemistry, Paderborn University, Warburger Str. 100, 33098 Paderborn, Germany; yuyang@mail.uni-paderborn.de (Y.Y.); sknust2@campus.uni-paderborn.de (S.K.); sschwid2@mail.uni-paderborn.de (S.S.); qqqqqqinqin@gmail.com (Q.Q.); ninayun716@gmail.com (Q.Y.); g.grundmeier@tc.uni-paderborn.de (G.G.)

* Correspondence: adrian.keller@uni-paderborn.de; Tel.: +49-5251-60-5722

XPS Characterization

In the survey spectra displayed in Figure S1 only oxygen, carbon, nitrogen, and titanium are present on the surfaces of all samples. Table S1 shows the quantification results based on XPS surveys measured at three different positions on each sample. All surfaces had very similar surface compositions, which were dominated by native absorbed carbon, titanium, and oxygen coming both from the titanium oxide layer and absorbed carbon.

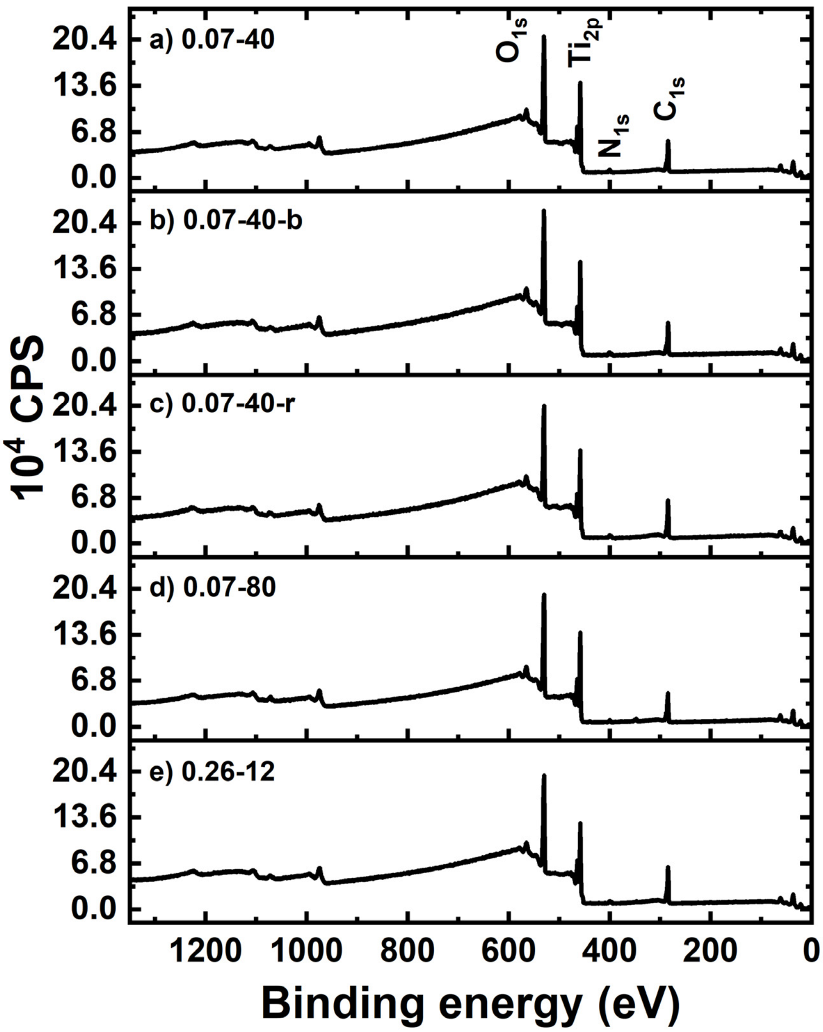

Figure S1. Ex-situ XPS survey of the various titanium coated substrates.

Table S1. XPS quantification results.

Sample ID	O 1s (at%)	C 1s (at%)	N 1s (at%)	Ti 2p (at%)
0.07-40	39.8 ± 1.8	40.3 ± 0.8	1.6 ± 0.5	18.3 ± 1.8
0.07-40-b	42.5 ± 0.2	37.8 ± 1.3	2.8 ± 0.5	16.9 ± 1.7
0.07-40-r	39.4 ± 0.8	43.1 ± 0.1	2.4 ± 0.2	15.1 ± 1.1
0.07-80	42.9 ± 0.4	36.3 ± 1.0	1.5 ± 0.1	19.5 ± 1.4
0.26-12	37.2 ± 1.0	42.7 ± 2.0	2.2 ± 0.1	17.9 ± 2.3

To investigate the composition of the titanium oxide, the Ti 2p spectra were evaluated (Figure S2). To this end, the spectra were fitted taking into account the possible titanium oxidation states related to metallic titanium and different oxide structures (Ti, TiO, Ti_2O_3, and TiO_2) as well as the respective peak splitting. The peak positions are 453.4 eV and 459.2 eV for Ti 0+, 455.0 eV and 460.4 eV for Ti 2+, 456.6 eV and 462.4 eV for Ti 3+ and 458.4 eV and 464.2 eV for Ti 4+. The fitting results are displayed in Table S2. It can be seen that all sample surfaces were mainly composed of TiO_2 and only show minor differences in the suboxide composition. Therefore, we can conclude that the different deposition conditions did have almost no effect on the surface composition.

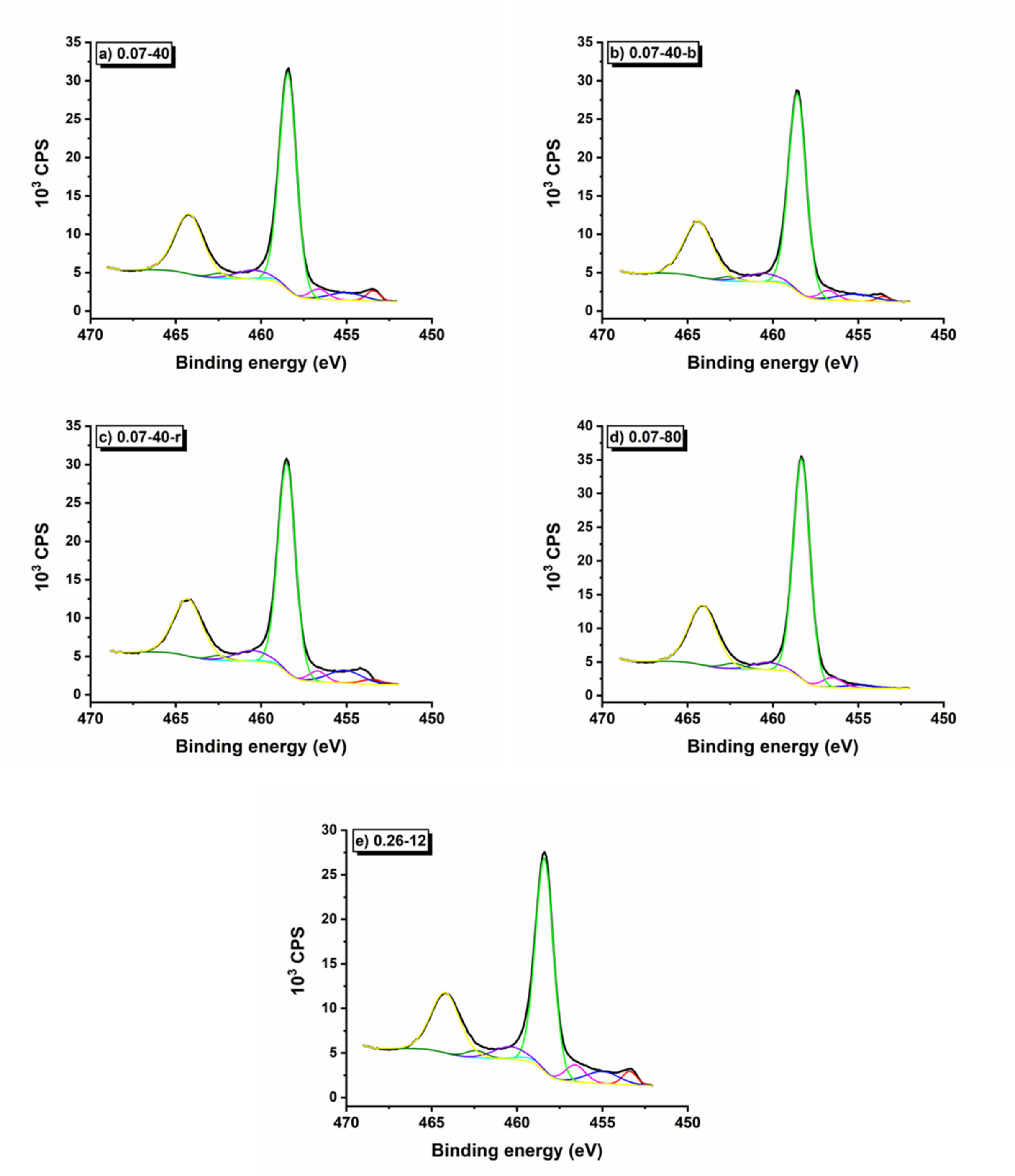

Figure S2. Ex-situ XPS Ti 2p high-resolution spectra of the various titanium coated substrates.

Table S2. Results of the Ti 2p deconvolution.

Sample ID	Ti 0+	Ti 2+	Ti 3+	Ti 4+
0.07-40	2.2 ± 1.6	6.7 ± 1.7	4.7 ± 0.4	86.3 ± 3.0
0.07-40-b	1.8 ± 0.2	8.7 ± 0.4	4.6 ± 0.4	84.9 ± 0.1
0.07-40-r	2.2 ± 0.3	12.6 ± 0.5	4.0 ± 0.2	81.2 ± 0.1
0.07-80	0.0 ± 0.0	5.1 ± 0.1	5.6 ± 0.2	89.4 ± 0.2
0.26-12	4.5 ± 0.6	10.5 ± 0.9	7.7 ± 1.4	77.2 ± 1.1

Morphological Surface Characterization

Table S3. Calculation of moment-based surface statistical parameters.

Parameter	Equation
RMS surface roughness	$Sq = \sqrt{\frac{1}{MN}\sum_{m=1}^{M}\sum_{n=1}^{N}(z(x_m, y_n) - \langle z \rangle)^2}$
Arithmetic surface roughness	$Sa = \frac{1}{MN}\sum_{m=1}^{M}\sum_{n=1}^{N}\lvert z(x_m, y_n) - \langle z \rangle \rvert$
Skewness	$Ssk = \frac{1}{Sq^3}\frac{1}{MN}\sum_{m=1}^{M}\sum_{n=1}^{N}(z(x_m, y_n) - \langle z \rangle)^3$
Kurtosis	$Sku = \frac{1}{Sq^4}\frac{1}{MN}\sum_{m=1}^{M}\sum_{n=1}^{N}(z(x_m, y_n) - \langle z \rangle)^4$

Table S4. Comparison of the fractal dimension determined by the cube counting method and from a linear fit to the slope of the power spectra in the log-log plot. Note that the linear fit was applied only to the correlated part of the power spectrum.

	0.07-40	0.07-40-b	0.07-40-r	0.07-80	0.26-12
Cube counting	2.48 ± 0.03	2.51 ± 0.03	2.49 ± 0.02	2.48 ± 0.01	2.51 ± 0.02
Power spectrum	1.16 ± 0.34	1.91 ± 0.18	1.13 ± 0.48	1.14 ± 0.36	1.22 ± 0.31

Exponential Fits of the Ellipsometry Data in Figure 2

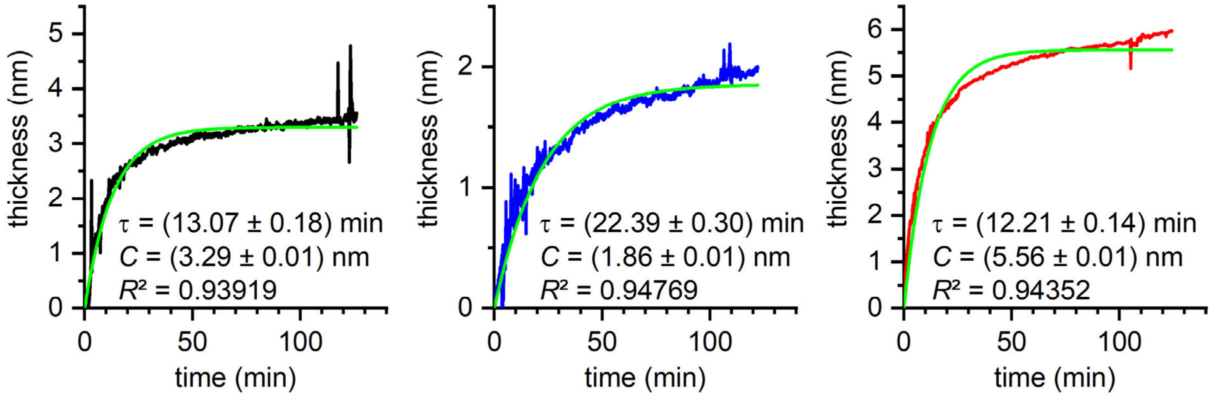

Figure S3. Protein layer thickness for MGB (left), BSA (center), and TGL (right) at surface 0.07-40 as measured by ellipsometry. The green lines are fits according to equation 1. The fit results are given in the plots.

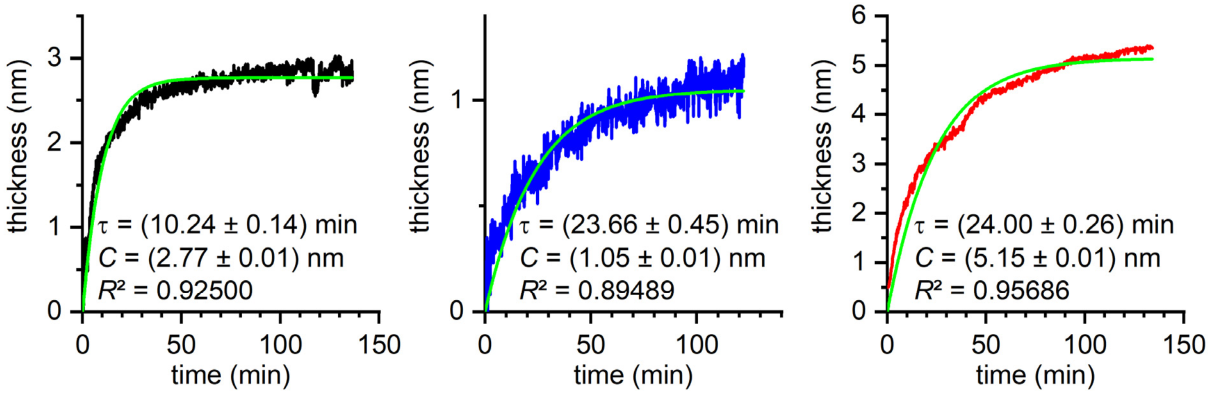

Figure S4. Protein layer thickness for MGB (left), BSA (center), and TGL (right) at surface 0.07-40-b as measured by ellipsometry. The green lines are fits according to equation 1. The fit results are given in the plots.

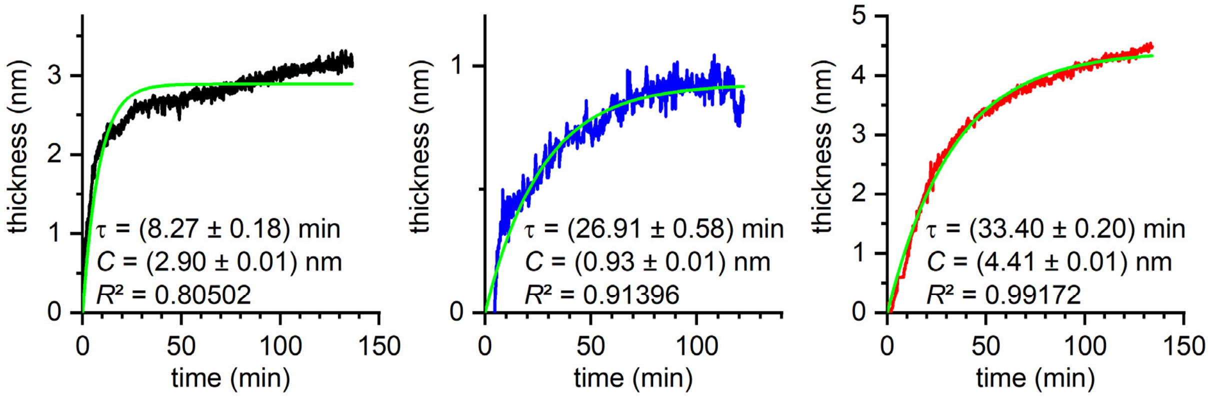

Figure S5. Protein layer thickness for MGB (left), BSA (center), and TGL (right) at surface 0.07-40-r as measured by ellipsometry. The green lines are fits according to equation 1. The fit results are given in the plots.

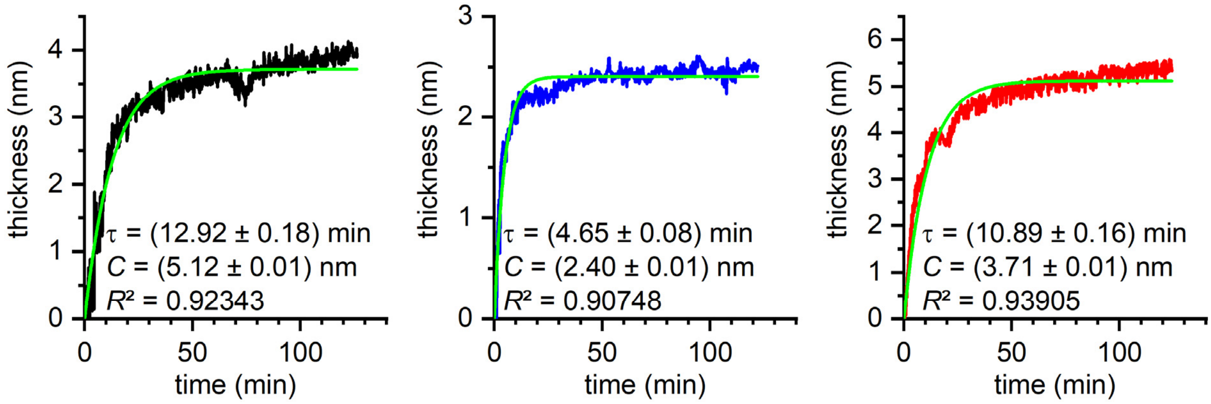

Figure S6. Protein layer thickness for MGB (left), BSA (center), and TGL (right) at surface 0.07-80 as measured by ellipsometry. The green lines are fits according to equation 1. The fit results are given in the plots.

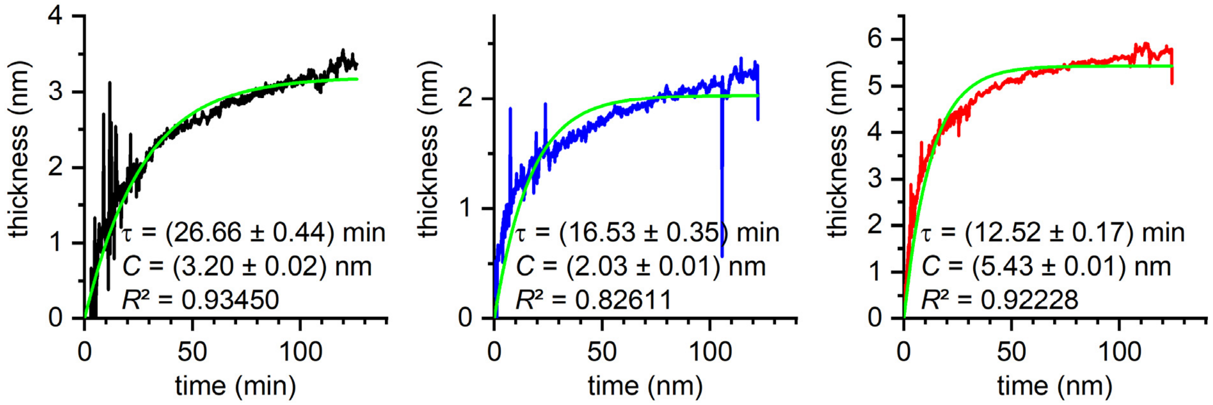

Figure S7. Protein layer thickness for MGB (left), BSA (center), and TGL (right) at surface 0.26-12 as measured by ellipsometry. The green lines are fits according to equation 1. The fit results are given in the plots.

R^2 Values of the Cross-Correlations

Table S5. R^2 values of the linear fits to the time constant data shown in Figure 3 of the main manuscript.

	MGB	BSA	TGL
Sq	0.20365	0.76429	0.25609
Sa	0.19994	0.75003	0.25081
R	0.29764	0.80876	0.36103
Ssk	0.70190	0.00234	0.72380
Sku	0.11679	0.46728	0.22706
D	0.00873	0.42884	0.04563

Table S6. R^2 values of the linear fits to the protein layer thickness data shown in Figure 5 of the main manuscript.

	MGB	BSA	TGL
Sq	0.68370	0.61157	0.00341
Sa	0.67248	0.60762	0.00311
R	0.68094	0.68123	0.03903
Ssk	0.15972	0.48051	0.86673
Sku	0.00042	0.00001	0.79996
D	0.38621	0.22932	0.06698
